# Cognitive function, balance, and physical activity among perimenopausal and postmenopausal women: a cross-sectional study

**DOI:** 10.3389/fgwh.2026.1830226

**Published:** 2026-06-15

**Authors:** Alaa M. Albishi, Jawahr Alagil, Sarah Aljunaini, Shaden Alsayyari, Renad Alrashid, Aljouharah Alshammari

**Affiliations:** Rehabilitation Health Sciences Department, College of Applied Medical Sciences, King Saud University, Riyadh, Saudi Arabia

**Keywords:** balance, BBS, cognitive function, menopause, physical activity level, physical performance, SPPB, women

## Abstract

**Objective:**

This study aimed to examine cognitive function, physical performance, balance, and physical activity among perimenopausal and postmenopausal women, and to explore the relationships among these domains.

**Methods:**

In this cross-sectional study, 220 women were equally allocated into perimenopausal and postmenopausal groups. Cognitive function was assessed using the Mini-Mental State Examination (MMSE), physical performance using the Short Physical Performance Battery (SPPB), and balance using the Berg Balance Scale (BBS). Physical activity levels were evaluated using the International Physical Activity Questionnaire (IPAQ). Analysis of covariance (ANCOVA) was performed to compare group differences while controlling for age and body mass index (BMI). Partial correlation analyses were conducted to examine associations among variables after adjustment for age, BMI, and menopausal status.

**Results:**

After controlling for age and BMI, no significant differences were observed between perimenopausal and postmenopausal women in MMSE [F(1,216) = 1.09, *p* = 0.297], SPPB [F(1,216) = 1.48, *p* = 0.226], or BBS scores [F(1,216) = 0.30, *p* = 0.583]. Similarly, physical activity levels did not differ significantly between groups (*p* > 0.05). Significant positive correlations were identified between MMSE and SPPB scores (r = 0.413, *p* < 0.001), MMSE and BBS scores (r = 0.136, *p* = 0.046), and SPPB and BBS scores (r = 0.455, *p* < 0.001). IPAQ scores were not significantly associated with cognitive or functional outcomes.

**Conclusions:**

Cognitive function, physical performance, and balance appear to be interrelated in midlife women. However, menopausal status alone was not independently associated with these outcomes after adjustment for age and BMI. These findings underscore the importance of integrated functional assessment strategies to support healthy aging in women during the menopausal transition**.**

## 1 Introduction

Women exhibit a substantially higher risk of Alzheimer's disease compared with men, with nearly double the lifetime prevalence and a disproportionately greater number of cases, which cannot be explained solely by longer lifespan (1, 2). Accumulating evidence indicates that the menopausal transition is a critical period during which neurobiological and physical changes accelerate. Declines in ovarian function and circulating estradiol during perimenopause and early postmenopause affect neural, musculoskeletal, metabolic, and emotional systems (3, 4).

Perimenopause refers to the transitional phase leading up to menopause, characterized by hormonal fluctuations and irregular menstrual cycles, whereas menopause is defined as the permanent cessation of menstruation for at least 12 consecutive months (3, 4). During this transition, perimenopause is marked by pronounced hormonal variability, while early postmenopause involves sustained elevations in follicle-stimulating hormone before stabilization (4). These hormonal shifts contribute to vasomotor symptoms, sleep disturbances, mood instability, changes in physical performance, and early cognitive difficulties, which are commonly experienced by midlife women (4–6).

Cognitive function and physical activity levels often decline during this transition due to reductions in estradiol that disrupt synaptic plasticity, brain metabolism, and neurotrophic signaling (7–9). Approximately sixty percent of women report cognitive symptoms, including forgetfulness, concentration difficulties, and slower processing speed, during perimenopause, reflecting noticeable changes in memory and executive functioning (4, 10). Simultaneously, women in midlife often exhibit declines in overall physical activity, muscular strength, endurance, and functional mobility (11, 12). Activity levels may decline further in women experiencing early menopause, who tend to show greater decreases in participation and greater increases in sedentary behavior compared with those entering menopause at typical ages (12, 13). Given that estradiol regulates muscle metabolism, energy expenditure, motivation for movement, and neuromuscular coordination (14–18), declining hormone levels may contribute to changes in both cognitive performance and engagement in physical activity.

In addition to hormonal changes, other factors may contribute to cognitive and physical alterations during the menopausal transition. Sleep disturbances, vasomotor symptoms, mood changes, fatigue, and reduced quality of life are commonly reported during this period and have been associated with poorer cognitive performance, lower physical activity participation, and reduced functional capacity (4–6, 11–13). Furthermore, metabolic and neuromuscular changes associated with menopause may contribute to declines in muscle strength, mobility, balance, and overall physical functioning (14–17).

Balance performance also demonstrates measurable declines during the menopausal transition and shows significant links to cognition (19, 20). A reduction in estradiol levels affects neuromuscular efficiency, proprioception, and sensorimotor integration, which, in turn, contributes to decreased balance stability (19–21). Furthermore, balance impairments are closely associated with executive functioning, visuospatial ability, and attentional control, highlighting shared neural mechanisms underlying both postural regulation and cognitive processing (22, 23). Additionally, vasomotor symptoms, mood alterations, and sleep disturbances, common consequences of estrogen withdrawal, are associated with poorer cognitive outcomes and structural brain changes (14–17). Together, these findings show that cognition, physical activity, and balance are interconnected domains influenced by hormonal, neurological, and behavioral changes during menopause.

Reduced estradiol disrupts brain metabolism, physical performance, and postural control, contributing to functional limitations and increased fall risk (14–26). Lower physical activity levels are associated with poorer cognitive function and emotional well-being, whereas regular activity provides protective effects during midlife (26, 27). Balance impairments further reflect broader neuromuscular and cognitive vulnerabilities that may diminish independence and mobility (22, 23).

Despite growing evidence linking cognitive function, physical performance, and balance during the menopausal transition, few studies have comprehensively examined these domains simultaneously in perimenopausal and postmenopausal women, and even fewer have explored how these variables interact (24–28). Therefore, this study aims to compare cognitive function, physical performance, balance ability, and physical activity levels between perimenopausal and postmenopausal women and to examine the interrelationships among these measures.

## 2 Methodology

### 2.1 Study population and procedures

This cross-sectional study examined differences in physical and cognitive function between perimenopausal and postmenopausal women. An *a priori* power analysis was conducted using G*Power (University of Düsseldorf, Düsseldorf, Germany). Based on a previous study (29) that reported substantial differences in balance performance, as measured by the Berg Balance Scale, between middle-aged and older women, the expected effect size was set at Cohen's d = 0.80, with an alpha level of 0.05 and a desired statistical power of 0.90. The analysis indicated that 34 participants were required per group (total *N* = 68). To enhance statistical precision, increase robustness, and account for potential incomplete responses, 110 participants were recruited for each group. Participants were voluntarily recruited through direct invitations at the King Salman Social Center and classified into either the perimenopausal group (*N* = 110) or the postmenopausal group (*N* = 110). Participants were classified into perimenopausal and postmenopausal groups based on self-reported menstrual history in accordance with established reproductive aging criteria (3). Perimenopause was defined as the transitional phase characterized by irregular menstrual cycles or changes in cycle length and extending until 12 months after the final menstrual period. Postmenopausal was defined as the absence of menstruation for at least 12 consecutive months. Inclusion criteria required that participants be Saudi women aged 40 years or older, able to read, understand, and communicate in Arabic, and without major medical conditions. Exclusion criteria included being non-Saudi, younger than 40 years, or reporting any medical condition that could affect cognitive, psychological, or physical function. The study received ethical approval from the Institutional Review Board at King Saud University (No. E-25-9999) and was conducted between 15 August 2025 and 25 December 2025 in accordance with the Strengthening the Reporting of Observational Studies in Epidemiology (STROBE) guidelines.

Data collection took place in a designated room at the King Salman Social Center to ensure a standardized environment for both groups. All research procedures and study objectives were clearly explained to participants before data collection, and written informed consent was obtained from each participant. Individuals were informed of their right to withdraw at any time and assured that all collected information would remain confidential. To protect participant privacy and minimize potential bias during analysis, each participant was assigned a unique identification code. All data collection forms and electronic files were stored in a secure, password-protected online folder throughout the study.

### 2.2 Outcome measures

#### 2.2.1 International physical activity questionnaire–short form (IPAQ-SF)

The International Physical Activity Questionnaire (IPAQ) short form was used to evaluate participants’ physical activity levels (30–32). This seven-item tool collects information on vigorous and moderate activities, walking, and sedentary behavior over the previous week, including activity related to transportation, household tasks, fitness, and sports. Each type of activity is assigned a metabolic equivalent (MET) value, and total weekly activity minutes are calculated by multiplying the daily duration (in minutes) by the number of days per week for walking, moderate-intensity activities, and vigorous-intensity activities performed in bouts of at least 10 minutes. Energy expenditure is then expressed in MET-minutes per week by multiplying the time spent in each activity category by its corresponding MET value. A MET represents the energy cost of an activity relative to resting metabolic rate. In the IPAQ classification system, moderate activity generally reflects about 30 minutes of moderate-intensity movement on most days, while vigorous activity corresponds to roughly an hour or more per day at a higher intensity. Participants who did not meet the criteria for either moderate or high physical activity levels were categorized as having a low activity level. The Arabic version of the IPAQ-SF has demonstrated strong reliability, with test–retest correlations ranging from 0.63 to 0.74 for specific activity components and 0.79 for total activity, as well as acceptable criterion validity compared with accelerometer-based energy expenditure (r = 0.61) (30). Because physical activity commonly fluctuates during perimenopause and postmenopause, the IPAQ provides a reliable tool for comparing activity patterns between groups.

#### 2.2.2 Berg balance scale (BBS)

Balance performance was assessed using the Berg Balance Scale (BBS), a widely used and validated clinical instrument that measures static and dynamic balance across a range of everyday functional activities (33–35). The scale includes 14 tasks that reflect common postural and mobility challenges, such as standing without support, rising from a seated position, reaching forward, retrieving an object from the floor, turning 360 degrees, transferring between positions, and stepping onto a stool. These tasks collectively assess multiple aspects of balance control, including postural stability, anticipatory and reactive adjustments, and the ability to maintain or restore equilibrium during movement. Each item is scored on a 0–4 scale, where 0 indicates an inability to attempt or complete the activity, and 4 indicates safe, independent performance, yielding a total score of 0-56, with higher scores indicating better balance. Score ranges are commonly interpreted as follows: 0–20 indicates poor balance and a high fall risk, 21–40 suggests moderate impairment, and 41–56 indicates good balance and a relatively low fall risk. The BBS is valued for its practicality, requiring minimal equipment and approximately 15–20 minutes to administer, making it suitable for clinical, community, and research environments. It demonstrates excellent psychometric properties, with inter-rater reliability reported at 0.97 (95% CI: 0.96–0.98) and intra-rater reliability at 0.98 (95% CI: 0.97–0.99) in individuals with balance impairments (33), and its sensitivity to detecting subtle changes in postural control makes it particularly appropriate for examining expected balance differences between perimenopausal and postmenopausal women.

#### 2.2.3 Short physical performance battery (SPPB)

Lower-body physical performance was evaluated using the Short Physical Performance Battery (SPPB), a widely used instrument that measures functional mobility, strength, and balance in adults (36, 37). The SPPB consists of three components: a series of balance tests performed in progressively challenging stances (side-by-side, semi-tandem, and tandem), a 4-meter gait-speed assessment that captures walking efficiency and motor control, and a repeated chair-stand test that reflects lower-limb strength, power, and endurance. Each component is scored on a scale from 0 to 4 based on the participant's ability to complete the task and the time required, yielding a total score of 0 to 12, with higher scores indicating better physical performance. The SPPB has demonstrated excellent psychometric properties, including strong internal consistency (Cronbach's *α* = 0.86), high item–total correlations, and well-established construct validity, supported by a one-factor structure explaining 78.8% of the total variance (37). Its sensitivity to age-related and health-related changes in mobility makes it particularly suited to populations experiencing functional transitions. Given that lower-body strength, gait speed, and postural control may be influenced by hormonal and physiological changes during the menopausal transition, the SPPB serves as a valuable tool for detecting and comparing functional differences between perimenopausal and postmenopausal women.

#### 2.2.4 Mini-mental state examination (MMSE)

Cognitive function was evaluated using the Mini-Mental State Examination (MMSE), a widely used clinical screening instrument that assesses multiple domains of cognitive functioning, including orientation to time and place, attention and concentration, short-term memory, language comprehension and expression, and basic visuospatial and problem-solving skills (38, 39). The MMSE consists of a structured set of items that collectively yield a maximum score of 30, with higher scores reflecting better cognitive performance and lower scores indicating potential impairment. It is brief, easy to administer, and sensitive to detecting broad cognitive changes. The MMSE is commonly used in clinical and research settings to identify early cognitive decline or differences between population groups. The Arabic version employed in this study has demonstrated strong psychometric properties, including excellent reliability and cultural appropriateness, with high internal consistency (Cronbach's *α* = 0.88) and strong test–retest stability (ICC = 0.96), supporting its validity for use among Arabic-speaking adults (38). These characteristics make the MMSE suitable for evaluating overall cognitive function in women undergoing menopausal transitions, a period during which many experience fluctuations in memory, attention, and processing speed. Given evidence that cognitive complaints and measurable cognitive changes can occur during both perimenopause and postmenopause, the MMSE provides a standardized, reliable, and clinically meaningful method for comparing cognitive performance between groups. As a global cognitive screening tool, the MMSE was considered appropriate for assessing overall cognitive function and comparing group-level differences in this study.

### 2.3 Statistical analysis

All statistical analyses were performed using IBM SPSS Statistics, Version 27.0.1 (IBM Corp., Armonk, NY, USA). Descriptive statistics were used to summarize demographic, clinical, and outcome variables for the study population. Categorical variables, including menopausal group (perimenopausal or postmenopausal), educational level, employment status, and presence of chronic diseases, were presented as frequencies (N) and percentages (%), while continuous variables such as age and BMI were summarized as mean ± standard deviation (SD). Between-group differences in MMSE, SPPB, and BBS scores were examined using analysis of covariance (ANCOVA), with menopausal status as the independent variable and age and BMI included as covariates. Group differences in categorical variables, including physical activity level classifications, were examined using Pearson's chi-square test. Associations among continuous outcomes, specifically cognitive function, physical performance, and balance, were examined using partial correlation analysis while controlling for age, BMI, and menopausal status. To account for multiple comparisons, p-values were adjusted using the Bonferroni correction method. All statistical tests were two-tailed, and a p-value < 0.05 was considered indicative of statistical significance.

## 3 Results

### 3.1 Demographic and clinical characteristics

A total of 220 women participated in the study, with equal representation of perimenopausal (*n* = 110;50.0%) and postmenopausal (*n* = 110;50.0%) participants. As shown in Table 1, postmenopausal women were older and had higher BMI compared to perimenopausal women. Regarding educational level, a higher proportion of perimenopausal women held a bachelor's degree (57.3%) or a postgraduate degree (23.6%) than postmenopausal women (36.4% and 7.3%, respectively), whereas lower educational levels were more common among postmenopausal women. Employment status also differed between groups, with most perimenopausal women being employed (87.3%), while postmenopausal women were more frequently retired (30.0%) or unemployed (46.4%). In terms of chronic health conditions, a higher proportion of perimenopausal women reported no chronic disease (58.2%), whereas postmenopausal women more commonly reported multiple chronic diseases (47.3%). The most common individual conditions in both groups included high cholesterol and hypertension. A history of falling was reported by 14.5% of perimenopausal and 15.5% of postmenopausal women. Previous surgeries were more common among postmenopausal women (74.5%) compared to perimenopausal women (62.7%), while assistive device use was low in both groups (2.7% and 5.5%, respectively) (Table 1).

**Table 1:** Demographic and clinical characteristics of perimenopausal and postmenopausal women (*N* = 220).

Variables	Category	Perimenopausal (*n* = 110)	Postmenopausal (*n* = 110)
Age (Years)		45.32 ± 3.50	59.39 ± 7.23
BMI		28.76 ± 4.78	30.56 ± 4.72
Educational Level	Bachelor	63 (57.3%)	40 (36.4%)
	Postgraduate	26 (23.6%)	8 (7.3%)
	High school	12 (10.9%)	22 (20.0%)
	Diploma	7 (6.4%)	13 (11.8%)
	Middle school	2 (1.8%)	13 (11.8%)
	Elementary	0 (0.0%)	6 (5.5%)
	Illiterate	0 (0.0%)	8 (7.3%)
Employment Status	Employed	96 (87.3%)	26 (23.6%)
	Retired	2 (1.8%)	33 (30.0%)
	Unemployed	12 (10.9%)	51 (46.4%)
Presence of Chronic Diseases	High cholesterol level	16 (14.5%)	19 (17.3%)
	Hypertension	8 (7.3%)	10 (9.1%)
	Diabetes	7 (6.4%)	5 (4.5%)
	Heart disease	2 (1.8%)	1 (0.9%)
	Other	8 (7.3%)	5 (4.5%)
	Multiple chronic diseases	5 (4.5%)	52 (47.3%)
	No chronic disease	64 (58.2%)	18 (16.4%)
Currently or Previously Experienced Falling	Yes	16 (14.5%)	17 (15.5%)
	No	96 (87.3%)	93 (84.5%)
Previous Surgeries	Yes	69 (62.7%)	82 (74.5%)
	No	41 (37.3%)	28 (25.5%)
Assistive Device Use	Yes	3 (2.7%)	6 (5.5%)
	No	107 (97.3%)	104 (94.5%)

### 3.2 Physical and cognitive functions between peri- and post-menopausal women

Analysis of covariance (ANCOVA) was conducted to examine differences in cognitive function, physical performance, and balance between perimenopausal and postmenopausal women while controlling for age and BMI. For cognitive function, the overall model for MMSE scores was statistically significant [F (3,216) = 6.67, *p* < 0.001, R^2^ = 0.085]. However, menopausal status was not significantly associated with MMSE scores after adjustment [F (1,216) = 1.094, *p* = 0.297, partial *η*^2^ = 0.005]. BMI was significantly associated with MMSE scores [F (1,216) = 6.25, *p* = 0.013], whereas age was not (*p* = 0.297). For lower-body physical performance, the overall model for SPPB scores was statistically significant [F (3,216) = 11.21, *p* < 0.001, R^2^ = 0.135]. Menopausal status was not significantly associated with SPPB scores after adjustment [F (1,216) = 1.48, *p* = 0.226, partial *η*^2^ = 0.007]. BMI was significantly associated with SPPB scores [F (1,216) = 11.28, *p* < 0.001], whereas age was not significant (*p* = 0.157). For balance performance, the overall model for BBS scores was statistically significant [F (3,216) = 23.03, *p* < 0.001, R^2^ = 0.242]. Menopausal status was not significantly associated with BBS scores after adjustment [F(1,216) = 0.30, *p* = 0.583, partial *η*^2^ = 0.001]. Both age [F (1,216) = 11.87, *p* < 0.001] and BMI [F(1,216) = 17.53, *p* < 0.001] were significantly associated with BBS scores. Adjusted group comparisons are presented in Table 2.

**Table 2:** ANCOVA-Adjusted comparisons between perimenopausal and postmenopausal women (*N* = 220).

Variable	Perimenopausal (Adj. Mean ± SE)	Postmenopausal (Adj. Mean ± SE)	F (df =1,216)	p-value	Partial *η*^2^
MMSE	28.71 ± 0.33	28.12 ± 0.33	1.094	0.297	0.005
SPPB	10.42 ± 0.26	9.87 ± 0.26	1.477	0.226	0.007
BBS	52.84 ± 0.43	52.45 ± 0.43	0.303	0.583	0.001

Adjusted means (± SE) from ANCOVA controlling for age and BMI. MMSE, Mini-Mental State Examination; SPPB, Short Physical Performance Battery; BBS, Berg Balance Scale; SE, standard error; BMI, body mass index.

Physical activity level, assessed using IPAQ, was compared between perimenopausal and postmenopausal women. The results showed no statistically significant difference in the distribution of physical activity levels between the two groups (*χ*^2^ = 0.356, *p* = 0.837). Among perimenopausal women, 31.8% had low physical activity, 40.9% had moderate activity, and 27.3% had high activity. Similarly, among postmenopausal women, 28.2% had low physical activity, 43.6% had moderate activity, and 28.2% had high activity. Overall, the proportions of participants across the three physical activity categories were comparable between the groups, indicating that physical activity levels did not differ meaningfully between perimenopausal and postmenopausal women (Table 3).

**Table 3:** Physical activity levels (iPAQ) by menopausal status (*N* = 220).

Group	IPAQ			Chi-Squared	P-Value
	Low	Moderate	High		
Peri-Menopausal	35 (31.8)	45 (40.9)	30 (27.3)	0.356	0.837
Post-Menopausal	31 (28.2)	48 (43.6)	31 (28.2)		

### 3.3 Correlation between cognition and physical functional performance

Partial correlation analysis controlling for age, BMI, and menopausal status demonstrated significant positive associations between cognitive function, physical performance, and balance. MMSE scores showed a moderate positive correlation with SPPB (r = 0.413**) and a weak but significant correlation with BBS (r = 0.136*), indicating that better cognitive function was associated with improved physical and balance performance. Additionally, SPPB and BBS were moderately correlated (r = 0.455**), reflecting consistency between physical performance and balance measures. In contrast, IPAQ-measured physical activity levels were not significantly associated with MMSE, SPPB, or BBS, indicating that self-reported physical activity was not related to cognitive or functional performance in this sample (Table 4).

**Table 4:** Partial correlations between cognitive function, physical performance, balance, and physical activity.

Variables	MMSE	SPPB	BBS	IPAQ
**MMSE**	1	0.413^b^	0.136^a^	0.094
**SPPB**	0.413^b^	1	0.455^b^	0.027
**BBS**	0.136^a^	0.455^b^	1	0.076
**IPAQ**	0.094	0.027	0.076	1

Values represent partial correlation coefficients (r) controlling for age, BMI, and menopausal status. Bonferroni correction was applied for multiple comparisons. MMSE, Mini-Mental State Examination; SPPB, Short Physical Performance Battery; BBS, Berg Balance Scale; IPAQ, International Physical Activity Questionnaire.

a *p* < 0.05.

b *p* < 0.001.

## 4 Discussion

### 4.1 Participant physical and cognitive characteristics

The characteristics of women in this study reflect common patterns seen in clinical practice among women transitioning through menopause. Our findings were aligned with known menopausal changes, where hormonal decline contributes to reduced metabolic efficiency and increased risk of chronic illnesses such as hypertension and diabetes (39–42). Additionally, the slightly higher frequency of falls and greater reliance on assistive devices observed in postmenopausal women align with evidence linking estrogen decline to decreased neuromuscular coordination, impaired proprioception, and lower balance stability (19–21, 25, 26).

On the other hand, no significant differences in physical function were observed between perimenopausal and postmenopausal women after adjusting for age and BMI. Specifically, SPPB and BBS scores were comparable between groups, suggesting that menopausal status alone may not influence lower-extremity physical performance or balance. The absence of significant differences after adjustment may reflect the close relationship between menopausal status and age, as age-related factors likely contribute to variations in balance performance, as evidenced by the significant association between age and BBS scores. Previous research has consistently shown that aging is associated with declines in postural control, proprioception, and neuromuscular coordination, all of which affect balance performance (19–21). In addition, BMI was significantly associated with both SPPB and BBS, suggesting that body composition may play an important role in physical function outcomes. Higher BMI has been associated with reduced mobility, impaired balance, and poorer physical performance due to increased mechanical loading and altered biomechanics. These findings are consistent with previous studies reporting that elevated BMI is linked to diminished functional capacity and an increased risk of mobility limitations among midlife and older adults (36, 37, 43). Overall, these findings suggest that variations in physical performance and balance are influenced more by age and BMI than by menopausal status alone. Although earlier studies have reported declines in balance and physical function across menopausal stages (11, 12, 21, 22), the present results indicate that these differences may be attenuated when key confounding factors are considered. From a clinical perspective, this highlights the importance of accounting for both age and body composition when evaluating mobility, balance, and fall risk in midlife women.

Similarly, cognitive performance did not differ significantly between perimenopausal and postmenopausal women after adjustment for covariates, suggesting that menopausal status alone may not independently influence global cognitive function as assessed by the MMSE. In contrast, BMI showed a significant association with MMSE scores, indicating that body composition and metabolic factors may play a more prominent role in cognitive performance within this sample. However, age was not significant in the adjusted model, suggesting that its influence may overlap with other variables, whereas BMI demonstrated a more consistent relationship with cognitive outcomes. These findings are consistent with previous evidence linking elevated BMI and metabolic dysregulation to poorer cognitive performance through mechanisms including inflammation, insulin resistance, and vascular alterations that affect brain function (44, 45). While prior studies have reported cognitive decline during the menopausal transition (4, 10), and neuroimaging research has identified menopause-related structural and functional brain changes (46–48), the present findings suggest that these effects may be subtle and influenced by broader physiological factors rather than menopausal status alone. Although some statistical associations were observed, the effect sizes were very small, suggesting limited clinical relevance of menopausal status alone on cognitive and functional outcomes. Furthermore, the MMSE is considered suitable for detecting global cognitive change; however, it may lack sensitivity to detect domain-specific cognitive changes, such as impairments in executive function or processing speed, which are more commonly affected during menopause.

Importantly, despite the absence of group differences, the significant positive associations observed among cognitive function, physical performance, and balance indicate that these domains remain closely interrelated. These findings are consistent with previous literature demonstrating that cognitive processes, including attention, executive function, and visuospatial abilities, play a critical role in balance control and mobility (22, 23). Even slight variations in cognitive function may influence dual-task performance, gait stability, and balance recovery, all of which are essential for fall prevention and functional independence (49).

Physical activity (PA) patterns provide important insight into behavioral changes occurring during the menopausal transition. In the present findings, the distribution of IPAQ classifications between perimenopausal and postmenopausal women was broadly similar, and the differences were not statistically significant. Participants in both groups were most frequently categorized as having moderate physical activity, with comparable proportions reporting low and high levels. These results suggest that overall self-reported physical activity levels were largely comparable between the two groups. Nonetheless, accumulating evidence indicates that middle-aged women generally experience a gradual decline in PA during the menopausal transition, characterized by reduced activity duration and frequency and increased sedentary behavior. Women who undergo menopause at an earlier age also tend to report lower PA levels compared with those transitioning at a typical age range (11–13). The progressive decline in estrogen associated with ovarian aging has been proposed as a contributing factor to these behavioral changes (14–18). Although IPAQ results are based on subjective self-report and may be influenced by recall and perception bias, the findings suggest that physical activity patterns in this sample remained relatively stable across menopausal stages. In addition, objective functional assessments did not demonstrate significant differences in balance performance between groups after adjustment for age and BMI. However, the observed associations among balance, cognition, and physical performance underscore the importance of multidimensional assessments for evaluating functional status in women during the menopausal transition.

### 4.2 Relationships among cognitive function, physical performance, and physical activity levels

A key finding of this study was the significant association between cognitive function and physical performance. Moderate correlations among MMSE, SPPB, and BBS scores indicate that cognitive status and physical function are closely interrelated. These findings are consistent with previous research demonstrating that balance control, gait adaptability, and safe mobility depend on cognitive processes such as attention, executive function, and visuospatial abilities (10, 22–27). In clinical settings, this emphasizes the importance of assessing cognitive function not only to evaluate mental status but also to identify individuals at increased risk of mobility limitations and falls. Furthermore, the moderate association between SPPB and BBS scores suggests that changes in physical performance may occur concurrently across multiple domains, including gait speed, lower-extremity strength, and balance control (33, 34, 36). Collectively, these findings highlight the importance of comprehensive physical performance assessments rather than reliance on a single measure.

In contrast, IPAQ scores were not significantly correlated with cognitive or objective physical performance outcomes. This is consistent with evidence indicating that self-reported physical activity does not always accurately reflect functional fitness or physiological capacity and should therefore be interpreted alongside objective measures (50). Clinically, these results support prioritizing performance-based functional assessments when evaluating risk factors and functional decline across different stages of the menopausal transition.

### 4.3 Study limitations

Several limitations should be considered when interpreting these findings. First, the cross-sectional design prevents causal inference regarding the relationships among menopausal status, cognitive function, and physical performance. Longitudinal studies are needed to better understand how these variables change over time during the menopausal transition. Second, physical activity level was assessed using the IPAQ, a self-reported instrument that is subject to recall and social desirability biases and reflects activity only over the previous seven days, which may not capture habitual or long-term physical activity patterns. In addition, physical activity analyses were not adjusted for potential confounding factors such as employment status and the presence of chronic diseases, which may influence activity patterns. Potential confounding variables such as hormone replacement therapy use, depressive symptoms, sleep quality, and socioeconomic status were also not assessed and should be considered in future studies. Third, global cognition was measured using the MMSE, which may not be sensitive enough to detect subtle cognitive changes that can occur during early menopausal stages. More comprehensive neurocognitive assessments, particularly those evaluating executive function and processing speed, may provide deeper insight. Finally, although the sample size was adequate, the study was conducted within a specific community context, which may limit the generalizability of the findings to women from different cultural, socioeconomic, or health backgrounds.

### 4.5 Implications for practice

The present findings highlight the importance of a comprehensive clinical approach for women undergoing the menopausal transition. Although no significant differences were observed between perimenopausal and postmenopausal women in cognitive function, physical performance, balance, or physical activity after adjustment for age and BMI, significant associations among cognitive function, physical performance, and balance suggest that these domains should be evaluated collectively rather than independently. Routine screening of mobility, balance, and cognitive status in midlife women may facilitate early identification of individuals at risk of functional decline and falls. Integrating cognitive screening tools, such as the MMSE, with objective performance-based assessments may provide a more comprehensive understanding of functional health during this transitional period. Additionally, combining self-reported physical activity measures with objective functional evaluations may help clinicians design targeted interventions to maintain mobility, prevent falls, and support healthy aging.

## 5 Conclusion

The findings of this study suggest that menopausal status alone is not independently associated with cognitive function, physical performance, or balance after adjustment for age and BMI. Instead, BMI and age-related factors appear to contribute more substantially to variations in functional and cognitive outcomes. Significant associations among MMSE, SPPB, and BBS scores indicate a close interrelationship between cognitive and motor domains, supporting the concept that functional health in midlife women is multidimensional. Furthermore, the lack of significant associations between IPAQ scores and objective functional measures suggests that self-reported physical activity may not fully reflect actual functional capacity. Overall, these findings support the use of integrated cognitive and physical performance assessments to promote early detection of functional limitations and support healthy aging in midlife women.

## Data Availability

The original contributions presented in the study are included in the article/Supplementary Material, further inquiries can be directed to the corresponding authors.
